# Supplementary material for: Non-dispersive infrared multi-gas sensing via nanoantenna integrated narrowband detectors
Source: Nat Commun. 2020 Oct 16;11:5245. doi: 10.1038/s41467-020-19085-1 (PMC7567865; doi:10.1038/s41467-020-19085-1)
Supplement: Supplementary file 2 — Supplementary Information [file 41467_2020_19085_MOESM2_ESM.pdf]

Supplementary Information

Non-dispersive infrared multi-gas sensing via nanoantenna integrated narrowband detectors

Xiaochao Tan[†], Heng Zhang[†], Junyu Li[†], Haowei Wan[†], Qiushi Guo[§], Houbin Zhu[△], Huan Liu[†], Fei Yi[,†]*

[†]School of Optical and Electronic Information and Wuhan National Research Center for Optoelectronics (WNLO), Huazhong University of Science and Technology, Wuhan, 430074, China

[§]Department of Electrical Engineering, Yale University, New Haven, Connecticut, 06511, USA

[△]School of Physics, Shandong University, Jinan, 250100, China

(*feiyi@hust.edu.cn)

Supplementary Note 1

The configuration of the conventional NDIR gas sensor

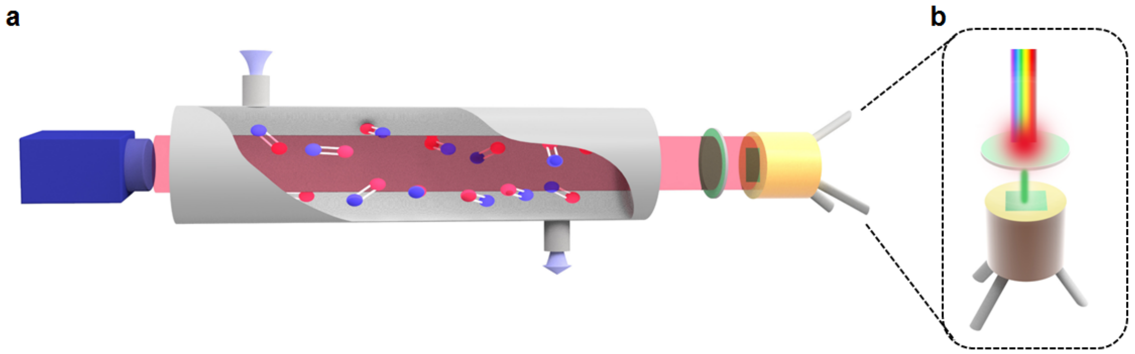

Supplementary Figure 1 | a, The configuration of the conventional NDIR gas sensor based on an infrared detector paired with an optical filter. The system is composed of four parts: light source, gas cell, optical filter, and detector. **b**, In conventional NDIR architecture, an optical filter in transmission mode is added before the detector to transmit the allowed wavelengths and reject unwanted wavelengths.

Supplementary Fig. 1a shows that the main components of the conventional NDIR gas sensor are a broadband infrared source (lamp), a sample gas chamber, a separate narrow bandpass filter that is paired with an infrared detector. The narrow bandpass filter providing the spectral selectivity is realized by a multilayer thin film stack deposited on a transparent substrate. Only wavelengths within a certain spectral range are allowed to pass through the filter and reach the detector, as shown in Supplementary Fig. 1b.

Supplementary Note 2

Design of plasmonic metamaterial absorbers for 8 target gases

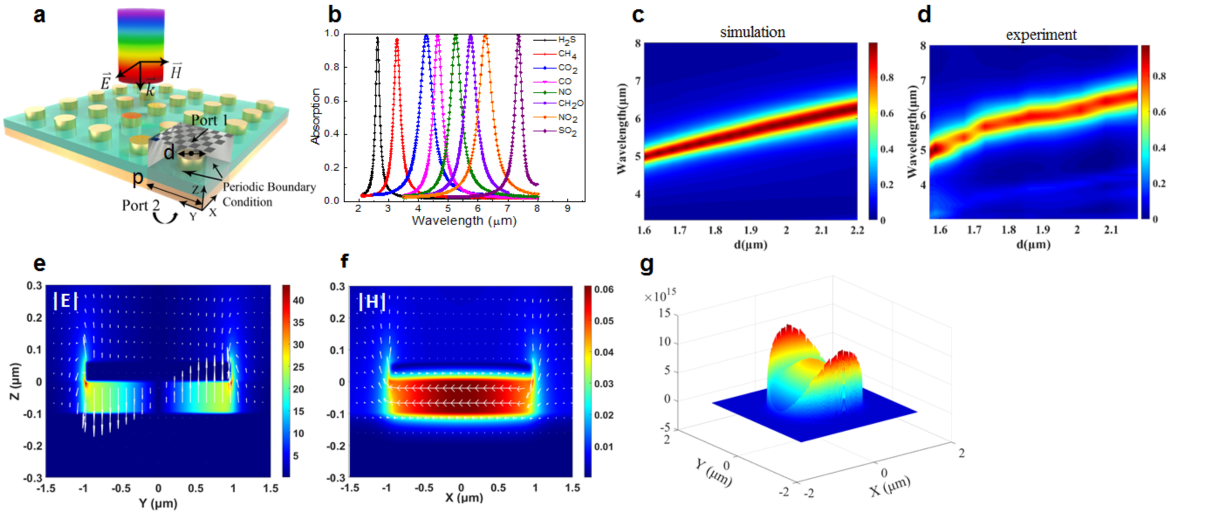

Supplementary Figure 2 | a, The configuration of the MIM absorber used in the optical numerical simulation. **b,** The simulated absorption spectra of 8 MIM absorbers that are correlated to the infrared absorption bands of 8 target gases, H_2S, CH_4, CO_2, CO, NO, CH_2O, NO_2, SO_2. **c-d,** The simulated and experimental spectral absorption as a function of the nanodisk diameter *d* with the lattice constant *P* =2 μm, respectively. **e,** The distribution of the electric field magnitude |**E**| and electric field vector **E** in the YZ cut-plane of a MIM absorber at its resonant wavelength λ_{peak} = 5.73 μm. **f,** The distribution of the magnetic field magnitude |**H**| and magnetic field vector **H** in the XZ cut-plane of the MIM absorber at λ_{peak} = 5.73 μm. **g,** The distribution of the electromagnetic loss density at the interface between the nanoantenna layer and the spacer at λ_{peak} = 5.73 μm.

Gas	Characteristic Absorption Wavelength [μm]	Diameter [μm]	Period [μm]	Medium Thickness [μm]
H_2S	2.64	0.726	2	0.08
CH_4	3.27	0.94	2	0.08
CO_2	4.26	1.29	2	0.08
CO	4.67	1.47	3	0.1
NO	5.26	1.72	3	0.1
CH_2O	5.73	2.06	3	0.1
NO_2	6.2	2.26	3	0.1
SO_2	7.35	3.4	4	0.11

Supplementary Table 1 | Design parameters of the MIM absorbers for 8 target gases

To optimize the infrared absorption characteristics of the plasmonic metamaterial absorber, we first used COMSOL, a finite element method based solver to numerically study the optical properties of the absorbers

employing periodic boundary conditions and plane wave excitation polarized along the y-axis (Cartesian coordinate system in Fig. 1(c)). Supplementary Fig. 2a shows the configuration of the absorber for parameter tuning: the diameter d of the nanodisk, the lattice constant (period) P of the array, the thickness t_{SiO2} of the silicon dioxide spacer. The thickness t_{disk} of the nanodisk and the thickness $t_{backplate}$ of the backplate are chosen to be 50 nm and 100 nm, respectively. Supplementary Table 1 is the relevant design parameters. The power transmission (T) and reflection (R) coefficients first calculated to determine the absorption efficiency defined as A = 1 − T − R. Supplementary Fig. 2b shows the simulated absorption spectra of 8 optimized MIM absorbers that are correlated to the infrared absorption bands of 8 target gases (H_2S, CH_4, CO_2, CO, NO, CH_2O, NO_2, and SO_2). As shown in Supplementary Fig. 2c-d, when P = 2 μm and t_{SiO2} = 80 nm (to fabricate multiple pixels on one single piece, so choose t_{SiO2} = 80 nm), the peak absorption wavelength λ_{peak} of the MIM absorber can be linearly tuned to cover the range from 2.5 μm to 5.5 μm by scaling the nanodisk diameter d. Supplementary Fig. 2e-f plot the local distribution of the electric field magnitude |**E**| and electric field vector **E**, the magnetic field magnitude |**H**| and magnetic field vector **H**, respectively, in a MIM absorber at its resonant wavelength λ_{peak} = 5.73 μm. Supplementary Fig. 2g plots the spatial distribution of the electromagnetic loss density at the interface between the nanoantenna layer and the spacer at λ_{peak} = 5.73μm.

Supplementary Note 3

Fabrication and packaging of the narrowband detector

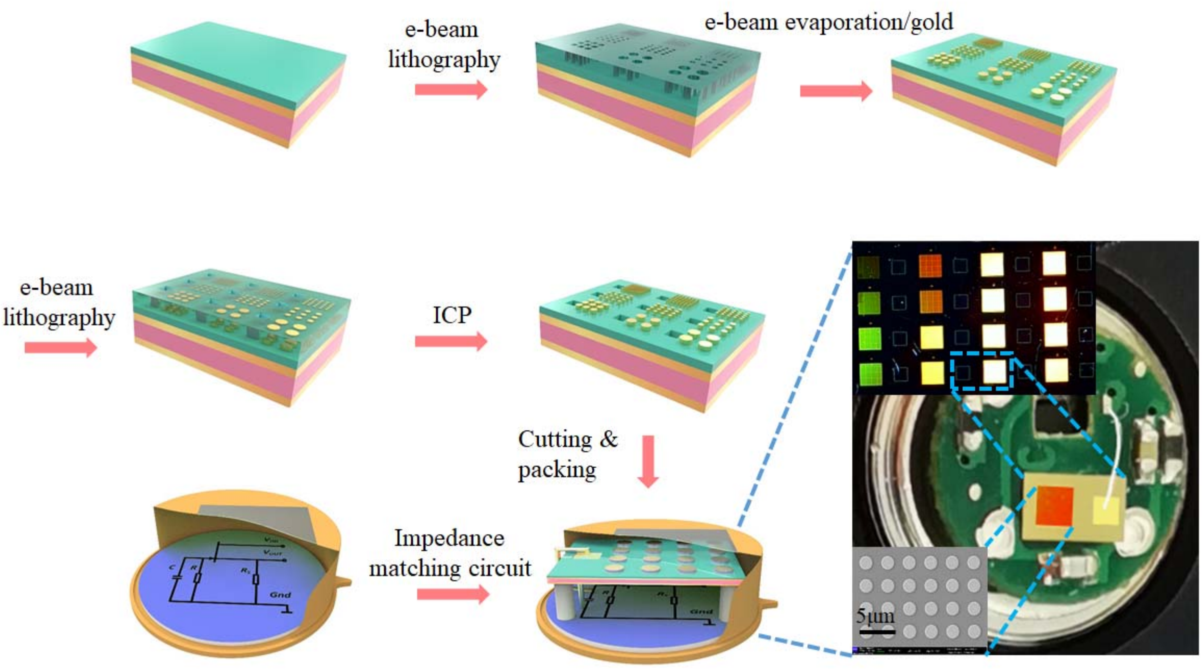

Supplementary Figure 3 | The fabrication and packaging of the narrowband detectors

Supplementary Fig. 3 presents the process of fabrication and packaging of the LT based narrowband detectors. The optical image of a TO-5 packaged single pixel narrowband detector, the optical image of an LT substrate with 16 MIM absorbers, and the SEM image of a nanodisk antenna array are also provided.

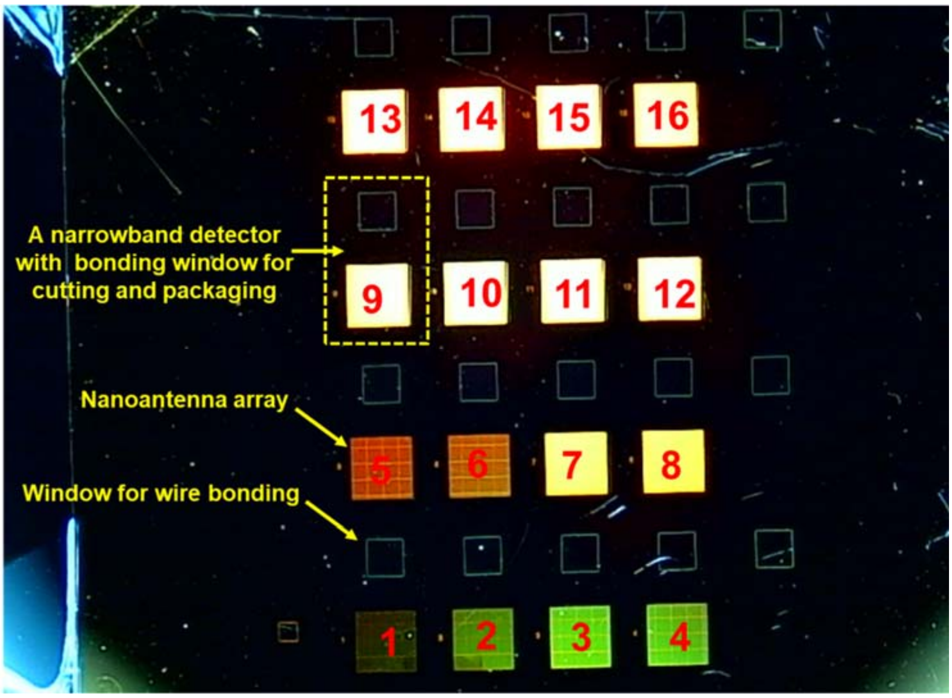

Supplementary Figure 4 | A group of 16 MIM absorbers fabricated on top of a 75um thick LT substrate

Supplementary Fig. 4 presents the optical microscope image of 16 MIM absorbers fabricated on top of a 75um thick LT substrate. Windows for wire-bonding and electrical connection are also created for each MIM absorber area. The area size of each absorber in this work is 1x1 mm. But it can be easily expanded to 2x2 mm, 5x5 mm or even larger. The MIM aborbers can be patterned on 6 inch LT substrate using UV lithogrpaphy (stepper). The narrowband detectors can be cut and packaged either as a group of single pixel detectors or as a multi-pixel detector.

Supplementary Note 4

Simulation of the temperature change of the detector

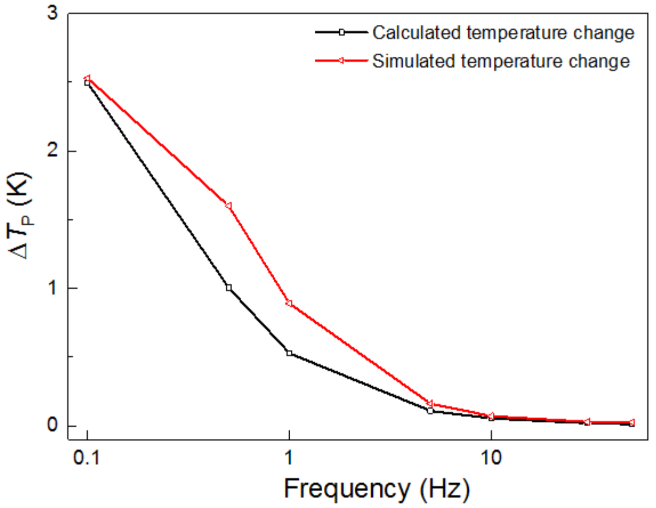

Supplementary Figure 5 | The simulated temperature change as a function of the modulation frequency of the optical chopper.

Material	Density ρ [g cm^{-3}]	Thermal Conductivity κ [W m^{-1} K^{-1}]	Pyroelectric Coefficient p [μc m^{-2} K^{-1}]	Permittivity ε_r	Specific Heat Volume c_p [J kg^{-1} K^{-1}]
$LiTaO_3$	7.45	4.6	230	54	423.93
Au	19.3	318			129
SiO_2	2.884	1.4			787

Supplementary Table 2 | Material properties used in the simulation

Since the pyroelectric current is generated when the temperature of the LT layer changes, the light source in the NDIR gas sensor needs to be modulated. A periodic square wave function is used to define a time-varying heat source in the heat transfer module of COMSOL. The frequency of the square wave function represents the modulation frequency of the optical chopper. A transient state study is then conducted to find out the temperature change $\Delta T_P \equiv T - T_0$ in the time domain, where T is the current temperature, and T_0 is the initial temperature. The numerically simulated temperature change ΔT_P as a function of the modulation frequency is plotted by the red line in Supplementary Fig. 5.

As a comparison, we also calculate the temperature change ΔT_P using the equation[1, 2]:

$$\widetilde{\Delta T_P} = \frac{\alpha\tau_F\widetilde{\Phi_S}}{G_T}\frac{1}{\sqrt{1+(\omega\tau_T)^2}} \tag{1}$$

Where $\widetilde{\Delta T_P}$ is the mean of the temperature change ΔT_P, α =0.82 is the absorption rate of MIM, $\tau_F = 1$ is the transmission rate of the window of the detector, $\widetilde{\Phi_S}$=3.512 mW is the source power, $G_T = 4 * \frac{W*(\kappa_1*t_1+\kappa_2*t_2+\kappa_3*t_3+\kappa_4*t_4)}{\mathrm{L}} = 1.022$ mW K^{-1} is the thermal conductance of multilayer, where W is the width of the each layer, κ_i is the thermal conductivity of each layer, t_i is the thickness of each layer, L is the length of each layer. ω is the angular frequency, τ_T=$\frac{H_P}{G_T}$ =0.834 s is the thermal time constant, which is little different from the simulated thermal time constant, where $H_P = c_p * \rho * A_d * t_p = 0.853$ mWs K^{-1}, c_p is the specific heat volume, ρ is the density, $A_d = 3.6$ mm is the plane area， $t_p = 75$ μm is the thickness of the lithium tantalate. The theoretically calculated temperature change ΔT_P as a function of the modulation frequency is plotted by the black line in Supplementary Fig. 5. It can be seen that the numerically simulated temperature change ΔT_P agrees well with the theoretical calculation using Supplementary Equation 1.

Supplementary Note 5

Theoretical calculation of the voltage response and noises of the detector:

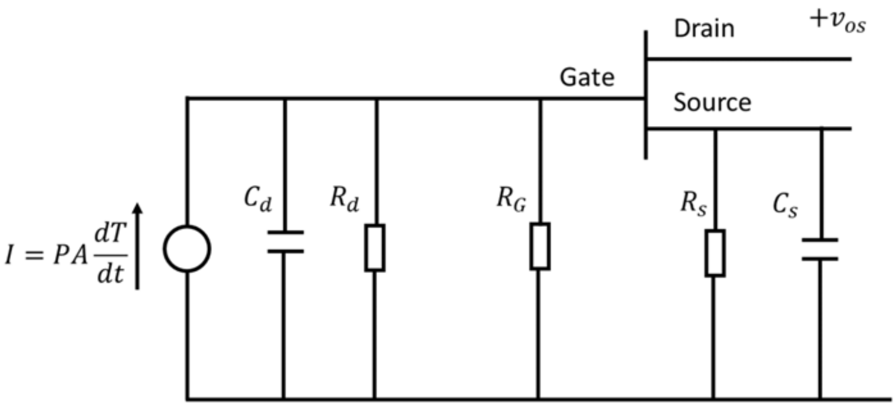

Supplementary Figure 6 | The circuit diagram of the packaged narrowband LT detector

Supplementary Fig. 6 presents the circuit diagram of the packaged narrowband LT detector. The LT detector is modelled as a parallel plate capacitor C_d with a loss resistance R_d. The generated pyroelectric current is modelled as a current source. And the output of the LT element is coupled to the input of an amplifier, characterized by an input resistance R_G, which is in parallel with the loss resistance. R_s and C_s are load resistance and capacitance.

a. Calculation of voltage response

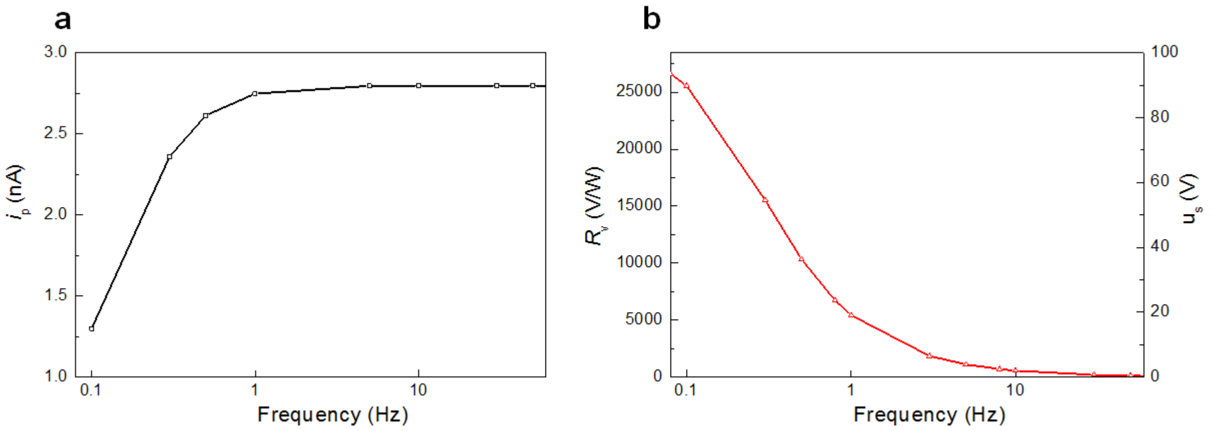

Supplementary Figure 7 | a, Pyroelectric current as a function of the modulation frequency of the optical chopper. **b**, Output voltage and voltage response as a function of the modulation frequency

To theoretically calculate the voltage response of the detector, we begin with the pyroelectric current caused by the temperature change:

$$\widetilde{i_p} = pA_d \frac{\widetilde{\Delta T_P}}{dt} \tag{2}$$

By plugging Supplementary Equation 1 into Supplementary Equation 2 we have:

$$\widetilde{i_p} = \omega p A_d \frac{\alpha \tau_F \widetilde{\Phi_S}}{G_T} \frac{1}{\sqrt{1+(\omega\tau_T)^2}} \tag{3}$$

where p=230 μc m^{-2} k^{-1} is the pyroelectric coefficient, $\omega = 2\pi f$ is the angular frequency. The theoretical value of $\widetilde{i_p}$ as a function of modulation frequency f is plotted in Supplementary Fig. 7a. It is seen that as the modulation frequency increases, the pyroelectric current rises first and then become saturated. When the modulation frequency is 5 Hz, the theoretically calculated temperature change is 0.11 K according to Supplementary Fig. 5 and the corresponding pyroelectric current obtained by Supplementary Equation 3 is 2.796 nA. After the impedance matching circuit, the voltage signal and voltage response can be calculated as follows:

$$U_s = \frac{pwA_d\alpha\tau_F\Phi_S * R}{G_T\sqrt{1+(w\tau_T)^2} * \sqrt{1+(wRC_d)^2}} \tag{4}$$

$$R_V = \frac{pwA_d\alpha\tau_F * R}{G_T\sqrt{1+(w\tau_T)^2} * \sqrt{1+(wRC_d)^2}} \tag{5}$$

where $R = \frac{1}{R_G} + \frac{1}{R_d} \approx 80\ \mathrm{G\Omega}$, is the total impedance, R_d= 80GΩ, R_G= 1TΩ, and $C_d = \frac{\varepsilon_0\varepsilon_r A_d}{t_p} = 22.95$ pF is the parallel plate capacitance of the LT element. As shown in Supplementary Fig. 7b, when the modulation frequency is 5 Hz, the voltage is 3.877 V, and the voltage responsivity is 1103.9 V W^{-1}. Note that in the main text, the average measured voltage response (90 V W^{-1}) is lower than the theoretically calculated value (1103.9 V W^{-1}). This can be mainly attributed to the fact that:

1) In the calculation, the LT is assumed to be supported by four Si posts that sit on the substrate (heat sink). The numerical model of the thermal analysis does not include the electrical connections of the LT element. While in experiment, the LT element was directly mounted on the printed circuit board containing the impedance matching circuit. Silver pastes are applied between the bottom electrode of the LT element and the pins on PCB to ensure good electrical connection. Also, the top electrode of the

LT element is wire bonded to the PCB for electrical connection. Thus, the heat conduction between the LT element and the PCB board of the packaged detector could be more significant than the simulated case.

2) In the calculation, the area size of the heat source A_s in the equation $i_p = pA_S \frac{\Delta T_P}{dt}$ is set to be the area size of the LT element (3.6 mm^2). While in the measurement, the spot size of the optical beam arriving at the LT element is about 0.8 mm. Therefore the size of the area heated up by the beam is only $\pi/4\times 0.8^2$ $mm^2 = 0.5024$ mm^2. This is another source of the difference between the calculated and measured voltage response.

b. Calculation of noise

The thermal fluctuation noise $\tilde{u}_{NT}$ and Johnson noise $\tilde{u}_{NR}$ are calculated using the following equations:

$$\tilde{u}_{NT} = \frac{R_V}{\alpha}(4kT^2 G_T \Delta f)^{1/2} \quad (6)$$

$$\tilde{u}_{NR} = \left(\frac{4kT}{R}\right)^{1/2} \frac{R}{[1+(\omega R C_d)^2]^{1/2}} A_V = \frac{\sqrt{\frac{4kT\Delta f}{R}}}{\omega c} \quad (7)$$

where R_V is the spectral voltage responsivity, k is the Boltzmann constant, T is the temperature, Δf is the noise bandwidth, A_V is the voltage gain(A_V ≈0.99). It is seen that the contribution of the Johnson noise generated by the loss resistance of the lithium tantalate is in general larger than the contribution of the thermal fluctuation noise.

Supplementary Note 6

The output power of the light sources as a function of wavelength

The details about the light sources used in the experiment can be found from the following webpages:

a) Globar IR source SLS203L/M from Thorlabs

https://www.thorlabs.com/newgrouppage9.cfm?objectgroup_id=7269&pn=SLS203L/M

b) Widely Tunable Mid-Infrared Quantum Cascade Laser LaserTune^{TM} from Block Engineering

https://www.blockeng.com/products/lasertune.html

The working wavelength of the widely tunable mid-IR QCL(LaserTune) can cover 5.4 µm – 12.8 µm if it is installed with all four laser sub-modules. In our measurement setup, the LaserTune QCL is only installed with 1 laser sub-module, so the working wavelength is from 5.4 µm – 6.0 µm

Supplementary Note 7

Absorption spectra of the MIM absorbers with full wavelength range

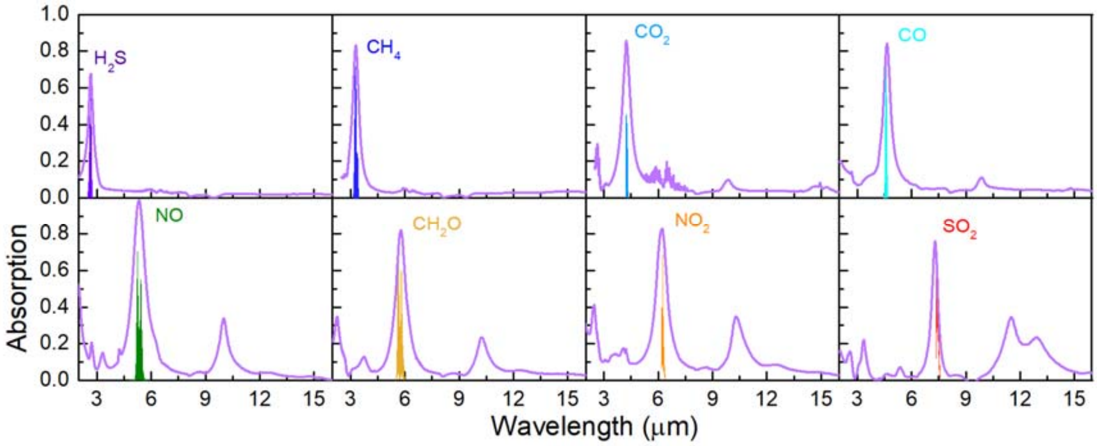

Supplementary Figure 8 | The measured absorption spectra of 8 fabricated MIM absorbers (purple solid lines) compared to the infrared absorption bands of 8 target gases: H_2S, CH_4, CO_2, CO, NO, CH_2O, NO_2, SO_2.

Supplementary Note 8

Comparison of the narrowband detectors with commercial LT detectors[3]

Reference	Thickness of LT element [μm]	Area size of LT element [mm]	Voltage responsivity Sv [V W^{-1}]	Specific detectivity D* [cm $Hz^{1/2}$ W^{-1}]	Noise equivalent power [nW/$Hz^{1/2}$]
Ref [3]	5	2×2	500	12×10^8	0.17
Ref [3]	20	2×2	540	8×10^8	0.25
This Work	75	2.4×1.5	90	1×10^8	19

Supplementary Table 3 | Comparison of the narrowband detectors with commercial LT detectors

Supplementary Table 3 compares the narrowband detectors with the commercial LT detectors (see Supplementary Ref [3]). It is seen that the performance of the narrowband detectors enabled by plasmonic metamaterial absorber developed in this work is comparable to the performance of the commercial LT detectors that use metal black coating as the IR absorber. Since the thickness of the LT element in this work (75 μm) is larger than the thickness of the LT element in Supplementary Ref [3] (20 μm and 5 μm) and the narrowband detectors in this work are not vacuum packaged, it is expected that the specific detectivity can be further improved by using thinner LT elements and implementing vacuum package.

Supplementary Note 9

The configuration of NDIR system

The gas cell used in this work is a White type multipass cell[4, 5] with an effective optical length of 5m. Its physical length is about 40 cm. Supplementary Fig. 9(a) illustrates the arrangement of the components in the NDIR system, including the Globar light source with the built-in beam collimating capability, the optical chopper, the multipass gas cell, the reflective objective and the narrowband detector that is mounted on an x-y-z translation stage. Supplementary Fig. 9(b) shows the optical configuration of White cell.

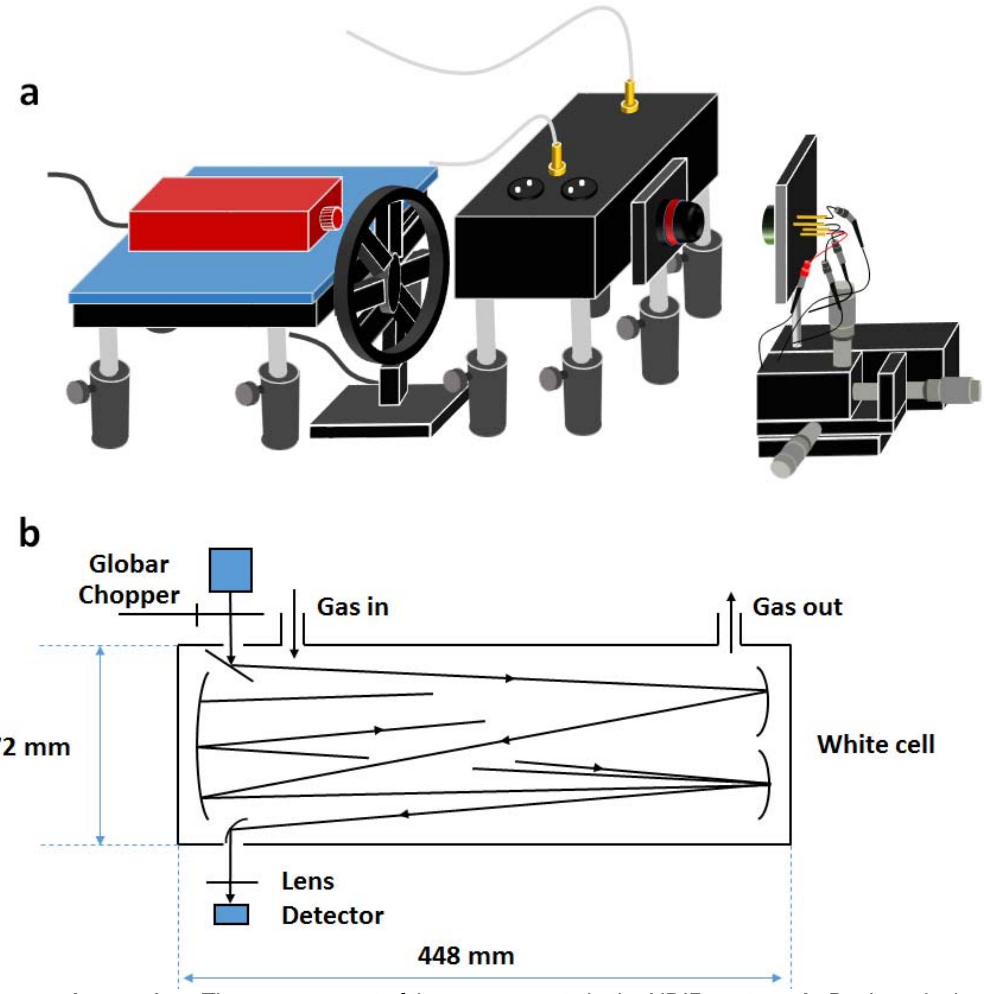

Supplementary Figure 9 | **a**, The arrangement of the compoments in the NDIR system. **b**, Basic optical configuration of the White cell.

The broadband IR source (SLS203L/M from Thorlabs) used in this work is a silicon carbide Globar light

source. The Globar is placed inside the housing, it is placed at one focus of an ellipsoid reflector. The output from the ellipsoid reflector is then collimated again with a CaF2 collimating lens.

More details about the output beam properties of SLS203L/M can be found at:
https://www.thorlabs.com/_sd.cfm?fileName=CTN002679-D02.pdf&partNumber=SLS203L/M

Note that in a commercial NDIR sensor such as CO_2 sensor, a dual-pixel detector is fitted with a pair of optical filters so that one filter is centered on 4260 nm as the detection channel and the other on 3900 nm as the reference channel. The concentration of CO_2 can be measured from the ratios of the output voltages of the two pixels. Measuring errors caused by dust or diminishing radiation intensity are removed by the use of the reference channel. While in our system, due to the high insertion loss caused by the gas cell, the power of the light that passes through the gas cell and arrives at the narrowband detector is on the order of μW and the corresponding output voltage is only a few mV. Thus we use a reflective objective to focus all the light onto the antenna array of the narrowband detector to ensure the output voltage is measurable. It is difficult to add a reference channel since this necessitates the split of the light in the gas cell and further reduce the optical power in the detection channel. Therefore we only implement the detection channel in the current system.

Supplementary Note 10

The calculation of the effective absorption coefficient

In the main text, the value of *k* in Equation 1 is calculated as followed[6, 7]:

$$\tau = \frac{I}{I_0} = \frac{\int_{\lambda_{min}}^{\lambda_{max}} S(\lambda)T(\lambda)k(\lambda)d\lambda}{\int_{\lambda_{min}}^{\lambda_{max}} S(\lambda)T(\lambda)d\lambda} = e^{-k_{eff} l_0 x_0} \tag{8}$$

and

$$k_{eff} = \frac{-\ln(\tau)}{l_0\, x_0} \tag{9}$$

Here k_{eff} represents the effective absorption coefficient of the target gas; I_0 is the optical length; x_0 is the gas concentration. The integration is from λ_{min} = 0.5 µm to λ_{max} = 9 µm.

$\boldsymbol{s(\lambda)} = \frac{\boldsymbol{2\pi h c^2}}{\boldsymbol{\lambda^5}} \frac{\boldsymbol{1}}{\boldsymbol{e^{hc/\lambda K T} - 1}}$ describes the spectral power of radiation from the globar IR source. It is the radiated power per unit area of emitting surface per unit wavelength at temperature *T*. *h* is the planck constant; *c* is the speed of light and *K* is the boltzman constant.

$\mathrm{T}(\lambda) = T_0 + \left(\frac{2A}{\pi}\right) \frac{w}{4(\lambda-\lambda_0)^2 + w^2}$ describes the spectral response of the narrowband detector. It is the lorentz fitting curve of the spectral absorption of the MIM absorber, where T_0 is an offset value, λ_0 is the center wavelength of spectral absorption, *w* is the full width at half the maximum, *A* is the area.

k(*λ*) represents the individual absorption lines of the target gas, extracted one by one from the HITRAN database www.spectraplot.com.

The calculated values of k_{eff} for the eight target gases and span, *c* for Fig. 5(b) are shown in Supplementary Table 4 below:

GAS	span	*k*	*c*
H_2S	0.56	$3.77574\text{x}10^{-09}$	1.24
CH_4	0.57	$2.42065\text{x}10^{-07}$	0.9
CO_2	0.55	$1.4564\text{x}10^{-06}$	0.87
CO	0.56	$2.69587\text{x}10^{-07}$	0.98

NO	0.56	$1.3898x10^{-07}$	0.98
CH_2O	0.56	$4.80283x10^{-07}$	0.96
NO_2	0.56	$1.90973x10^{-06}$	0.66
SO_2	0.56	$1.30559x10^{-06}$	0.9

Supplementary Table 4 | The parameters used to draw the red dashed lines in Fig. 5(b)

In practice, the parameters span and *c* are fitting parameters used to match the fitting curves to the measured data as close as possible. They are adjusted to account for the changes in experimental conditions due to the mounting and unmounting of narrowband detectors in each round of measurement.

Supplementary Note 11

Mixed gas experiment

To turn the problem around and see whether our model allows the calculation of the gas concentrations **x_1 and x_2** from the detector responses **D_1 and D_2**, we first write a computer program based the following mathematical model.

$$D_1 = \text{span}_{11} * \left(e^{-k_{11} l x_1^{c_{11}}}\right) + \text{span}_{12} * \left(e^{-k_{12} l x_2^{c_{12}}}\right) \quad (10)$$

$$D_2 = \text{span}_{21} * \left(e^{-k_{21} l x_1^{c_{21}}}\right) + \text{span}_{22} * \left(e^{-k_{22} l x_2^{c_{22}}}\right) \quad (11)$$

The values of the coefficient span_{ij}, k_{ij}, c_{ij} are listed in the following table:

Configuration of the experiment	Value of i and j	span_{ij}	k_{ij}	c_{ij}
Detector I<br>Fixed SO_2, changed CO	i = 1, j = 1	0.31	2.69587×10^{-7}	0.99
Detector I<br>Fixed CO, changed SO_2	i = 1, j = 2	0.99	3.89657×10^{-8}	0.84
Detector II<br>Fixed SO_2, changed CO	i = 2, j = 1	0.99	3.56494×10^{-8}	0.86
Detector II<br>Fixed CO, changed SO_2	i = 2, j = 2	0.23	1.30559×10^{-6}	0.86

Supplementary Table 5 | The parameters used to draw the red dashed lines in Fig. 6(b)

We then define $\Delta D_1 = |D_{1\text{-calculated}} - D_{1\text{-measured}}|$ and $\Delta D_2 = |D_{2\text{-calculated}} - D_{2\text{-measured}}|$, where $D_{1\text{-calculated}}$ and $D_{2\text{-calculated}}$ are the calculated detector responses, and $D_{1\text{-measured}}$ and $D_{2\text{-measured}}$ are the measured detector responses. Therefore, ΔD_1 and ΔD_2 represent the absolute difference between the the calculated response and measured response of the two detectors. We further define $\Delta D = \sqrt{\Delta D_1^2 + \Delta D_2^2}$ as the standard deviation of ΔD_1 and ΔD_2.

To work out the best values of the gas concentrations x_1 and x_2 for the given $D_{1\text{-measured}}$ and $D_{2\text{-measured}}$, the program initiates a two-level iteration that varys both x_1 and x_2 from 1 ppm to 12500ppm, with a step size of 2 ppm. In each iteration, the program finds the calculated detector responses $D_{1\text{-calculated}}$ and $D_{2\text{-calculated}}$ from the combinations of x_1 and x_2 using Supplementary Equation 10 and Supplementary Equation 11, and

then calculate ΔD_1, ΔD_2 and ΔD respectively. Finally, the program selects the combination of x_1 and x_2 that corresponds to the minimized ΔD (ΔD_{min}) as the best values for the given $D_{1\text{-measured}}$ and $D_{2\text{-measured}}$.

We select five cases from the mixed gas experiments, as shown by the measured data points A, B, C, D, and E in Supplementary Fig. 10, to implement the program. The experimental input gas concentrations, the measured detector responses, the minimized ΔD (ΔD_{min}), and the calculated gas concentrations are listed in Supplementary Table 6.

Ideally, if all the measured detector responses (purple squares) strictly follow the red dash lines in Supplementary Fig. 10, the calculated concentrations shall be the same as the experimentally used concentrations. However, it is seen that there are discrepencies between the calculated concentrations and the experimental concentrations. For example, case A in Supplementary Fig. 11, the experimental concentrations are x_1=10000 ppm and x_2=7500 ppm, while the calculated concentrations are x_1 = 10645 ppm and x_2 = 6691 pm. The discrepencies arise from the fact that the red dash lines in Supplementary Fig. 10 generated by Supplementary Equation 10 and Supplementary Equation 11 do not perfectly match the measured data points (detector responses). The physical causes of this mismatch include the instability of the output power from the IR source, the temperature drift of the detector responses and other changes in the measurement conditions during the NDIR experiments. We expect that by improving the measurement conditions of the NDIR experiment to ensure that the measured detector responses exactly match the red dash lines, the program can accurately work out the gas concentrations from the measured detector responses.

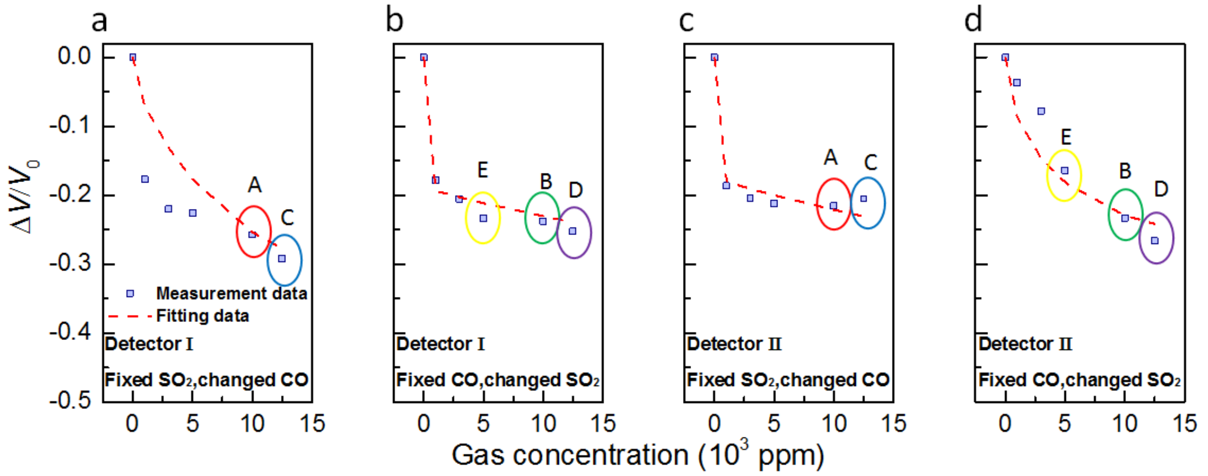

Supplementary Figure 10 | The detector responses of four mixed gas experiments: fixed SO_2 concentration and varying CO concentration measured by detector I; fixed CO concentration and varying SO_2 concentration measured by detector I; fixed SO_2 concentration and varying CO concentration measured by detector II; fixed CO concentration and varying SO_2 concentration measured by detector II, respectively. (in the maintext Fig. 6(b))

Experimental concentrations			Measured responses and minimized ΔD					Calculated concentrations	
Gas mixture	CO x_1 ppm	SO_2 x_2 ppm	$D_{1\text{-measured}}$	ΔD_1	$D_{2\text{-measured}}$	ΔD_2	ΔD_{min}	CO x_1 ppm	SO_2 x_2 ppm
A	10000	7500	-0.25721	0.00378	-0.21556	0.00537	0.00657	10645	6691
B	7500	10000	-0.2379	0.00766	-0.23291	0.00378	0.00854	7943	10337
C	12500	7500	-0.2919	0.01471	-0.20499	0.02568	0.02959	12499	5735
D	7500	12500	-0.25244	0.01352	-0.26633	0.02514	0.02854	8935	12499
E	7500	5000	-0.23335	0.02194	-0.16414	0.01768	0.02818	9653	3453

Supplementary Table 6 | The parameters of gas mixture A,B,C,D,E

Another way to determine the values of x_1 and x_2 for the given $D_{1\text{-measured}}$ and $D_{2\text{-measured}}$, is to set a certain error range of ΔD_1 and ΔD_2, and search for the combinations of x_1 and x_2 whose corresponding ΔD_1 and ΔD_2 are within the error range. For example, Supplementary Fig. 11 plots the color map of ΔD as a function of x_1 and x_2 with the corresponding $\Delta D_1 \leq 0.015$ and $\Delta D_2 \leq 0.015$. Each of these combinations of x_1 and x_2 in the color map can be regarded as a reasonable choice for the computer deduced x_1 and x_2.

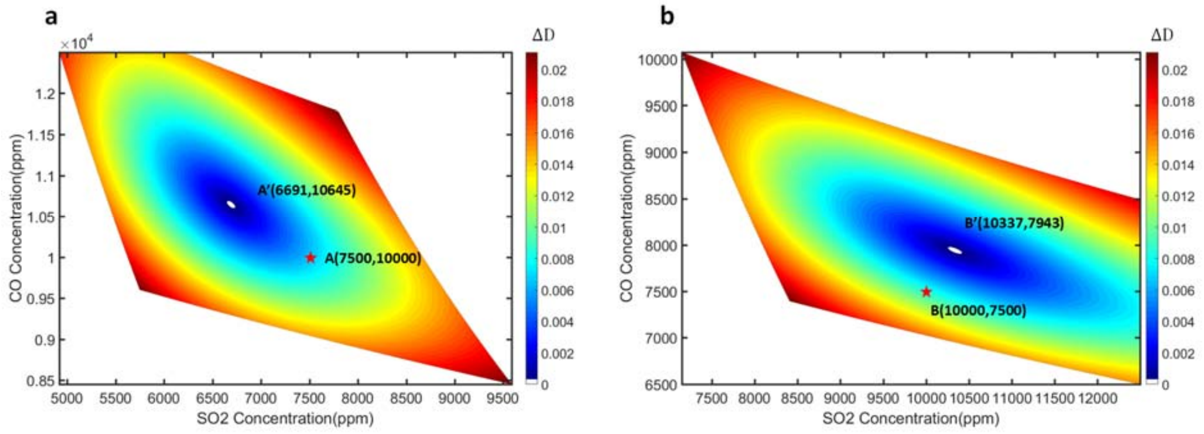

Supplementary Figure 11 | The color map of ΔD as a function of x_1 and x_2 with the corresponding $\Delta D_1 \leq 0.015$ and $\Delta D_2 \leq 0.015$ for **a,** gas mixture A, and **b**, gas mixture B. The red pentagram indicates the experimentally used x_1 and x_2. The white patch indicates the combinations of x_1 and x_2 with the corresponding $\Delta D \rightarrow 0$

Supplementary Note 12

The detection limit of eight target gases in single target gas measurement

The detection limits of the eight target gases calculated from the measured voltage responses in Fig. 5(b) are summarized in Supplementary Table 7. We calculate the gas detection limit C_{limit} using the following equation[8]:

$$C_{limit} = \frac{3\sigma\Delta C}{\Delta V} \quad (12)$$

where σ is the voltage deviation at 0 ppm (100% nitrogen atmosphere), and the $\Delta V/\Delta C$ is the sensitivity of the detector at low concentrations.

GAS	H_2S	CH_4	CO_2	CO	NO	CH_2O	NO_2	SO_2
Detection Limit [ppm]	489	63	2	11	17	27	54	104

Supplementary Table 7 | The detection limit of eight target gases in single target gas measurement

The level of cross-response for each of the neighboring gases on the spectrum is evaluated using $X_{MIN} = \frac{1}{k_{eff}l}\ln(\frac{V_0}{V_0-\Delta V_{MIN}})$, where ΔV_{MIN}=3*σ. The calculate values of X_{min} for detector I to VIII are summarized in Supplementary Table 8. For each detector, the X_{min} of its own target gas and the neighboring gases are provided. For example, for detector III that is used for CO_2, the X_{min} of CH_4, CO_2, and CO are provided.

**Detector**	**I (for H_2S)**		**II (for CH_4)**			**III (for CO_2)**			**IV (for CO)**		
Gas	H_2S	CH_4	H_2S	CH_4	CO_2	CH_4	CO_2	CO	CO_2	CO	NO
X_{min} (ppm)	3089	322	30114	89	106	149	4	28	41	121	806
**Detector**	**V (for NO)**			**VI (for CH_2O)**			**VII (for NO_2)**			**VIII (SO_2)**	
GAS	CO	NO	CH_2O	NO	CH_2O	NO_2	CH_2O	NO_2	SO_2	NO_2	SO_2
X_{min} (ppm)	101	50	19	81	13	7	89	18	381	54	9

Supplementary Table 8 | The level of cross-response for each of the neighboring gases on the spectrum

Supplementary Note 13

Thermal cross-talk

In our proposed architechture, each MIM area corresponds to a narrowband detection element. When multiple MIMs are built onto the same LT substrate, each MIM absorbs a certain portion of the incident light and dissipates the absorbed optical energy into heat. Thus the heat generated in one MIM area can affect the temperature of the neighbouring MIMs via thermal conduction and this is the thermal cross-talk.

To minimize the thermal cross-talk, the basic idea is to thermally isolate each narrowband detection element using its own heat sink. For example, the current 75μm thick LT substrate with built-in MIMs can be cut into separate narrowband detection elements. The separate detection elements can then be mounted on a PCB board via the pins that provide electrical connections, as shown in Supplementary Fig. 12. In this case the pins provide heat conduction to the PCB boards and thermally isolate each detection elements.

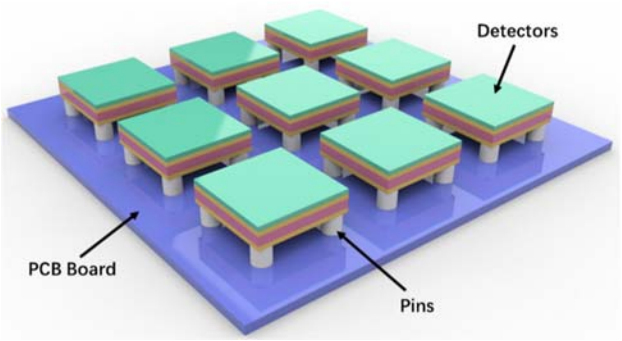

Supplementary Figure 12 | Packaging and thermal isolation of narrowband detectors using a PCB board

We also plan to fabricate MIMs on 700 nm thick LT thin film on silicon substrate (LTOI). To create themal isolation, one can fabricate deep trenches in the LT substrate between adjacent MIMs to reduce the thermal conduction, as shown in Supplementary Fig. 13.

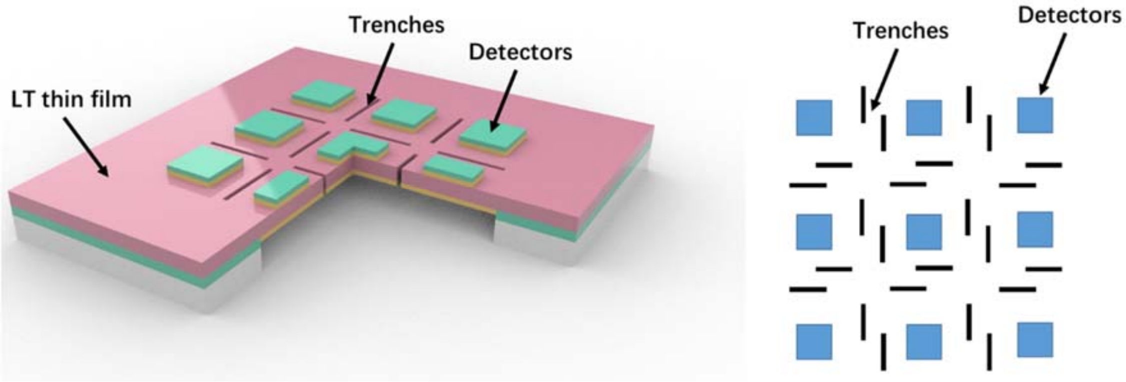

Supplementary Figure 13 | Thermal isolation of thin film LT based narrowband detector array using ICP-etch trenches

Supplementary References

1. Stuart B, "Infrared Spectroscopy: Fundamentals and Applications," John Wiley& Sons, Ltd., 2004.

2. Hossain A and Rashid M-H, "Pyroelectric Detectors and Their Applications," Ieee Transactions On Industry Applications, 27, 5 (1991).

3. Chatard J-P, Norkus V, Dennis PNJ. Pyroelectric infrared detectors based on lithium tantalate: state of art and prospects. **5251**, 121 (2004).

4. WHITE JU. John U. White, "Long Optical Paths of Large Aperture," J. Opt. Soc. Am. 32, 285-288 (1942). (1942).

5. Claude Robert, "Simple, stable, and compact multiple-reflection optical cell for very long optical paths," Appl. Opt. 46, 5408-5418 (2007), doi:10.1364/AO.46.005408.

6. Aleksandrov SE, Gavrilov GA, Kapralov AA, Matveev BA, Sotnikova GY, Remennyi MA. Simulation of characteristics of optical gas sensors based on diode optopairs operating in the mid-IR spectral range. *Technical Physics* **54**, 874-881 (2009).

7. Privalov VE*, et al.* Portable optoelectronic gas sensors operating in the mid-IR spectral range (λ=3 5 μm). **4680**, 188 (2002).

8. Sklorz A, Janßen S, Lang W. Detection limit improvement for NDIR ethylene gas detectors using passive approaches. *Sensors and Actuators B: Chemical* **175**, 246-254 (2012).
